# Size-Tunable Band Structure and Optical Properties of Colloidal Silicon Nanocrystals Synthesized via Thermal Disproportionation of Hydrogen Silsesquioxane Polymers

**DOI:** 10.1021/acs.jpcc.4c01462

**Published:** 2024-06-17

**Authors:** David S. Pate, Griffin C. Spence, Lisa S. Graves, Indika U. Arachchige, Ümit Özgür

**Affiliations:** † Department of Electrical and Computer Engineering, Virginia Commonwealth University, Richmond, Virginia 23284-9052, United States; ‡ Department of Chemistry, Virginia Commonwealth University, Richmond, Virginia 23284-9059, United States

## Abstract

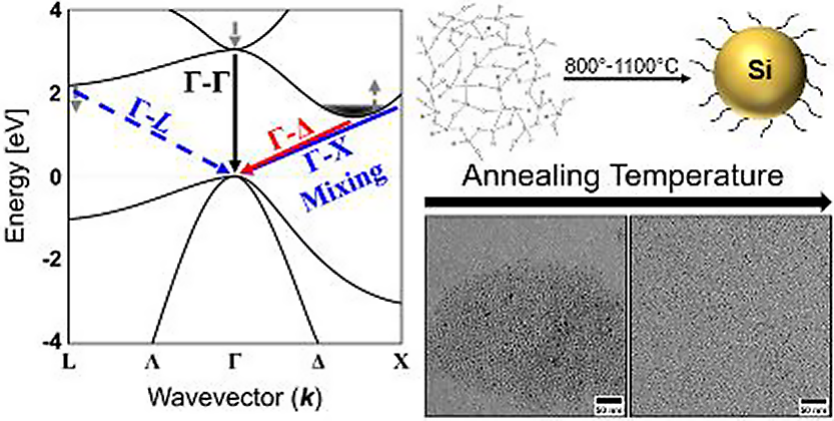

Dodecane-capped silicon nanocrystals (NCs) were synthesized by using a low-temperature (800–1100 °C) polymer variant of traditional hydrogen silsesquioxane thermal disproportionation. Highly crystalline Si NCs having tunable diameters (3.0–6.7 nm) and thus photoluminescence (PL) peaks (1.68–1.29 eV) were attained via changes in the maximum annealing temperature. Modifications in the NC band structure with diameter were explored by comparison of emission with absorption spectra obtained from diffuse reflectance spectroscopy. Large apparent energy shifts between onsets and PL were noted, being significant for smaller NCs (≤∼4.0 nm). This, along with comparatively “softer” onsets, is commensurate with density of states elongation around PL peaks associated with increasing confinement predicted for indirect semiconductor nanostructures. Tauc analyses of absorption additionally revealed three distinguishable optical transitions in all NCs: attributed to indirect Γ_25′_-Δ_1_ in lower energy ranges (likely the emission progenitor), indirect Γ_25′_-L_1_ overtaken by quasi-direct Γ-X wave function mixing for NC diameters ≤∼4.0 nm within the midenergy regime, and direct Γ_25′_–Γ_15_ transitions at energies nearing and above ∼3 eV.

## Introduction

The recent advent of silicon photonics has brought an exhibition of numerous nanocrystalline quantum dot (ncQD) structures having a wide variety of demonstrated applications in diverse fields, such as nanocrystal (NC)/QD-augmented light-emitting diodes (QD-LEDs) with quantum yields approaching ∼90% in consumer-facing devices^1−7^ and nontoxic and biocompatible *in*/*ex vivo* biological imaging agents.^8−13^ Although a significant number of techniques exist to fabricate Si nanostructures with tunable properties,^6,14−16^ high-temperature disproportionation of hydrogen silsesquioxane (HSQ) has been shown to reliably produce Si NCs having favorable physical and optical properties, namely high crystallinity and size-tunable core emission. Typically, this inherent size tunability is demonstrated by either controlling the cross-linking density of HSQ polymers via the volume of methanol used in its creation^17^ or changing the maximum annealing temperature during synthesis.^15,18−20^ To circumvent the limited commercial availability of HSQ and requirements for processing at temperatures that can exceed 1100 °C, various low-cost HSQ synthesis routes and annealing at relatively low temperatures (800–1100 °C) just below the melting point of Si have recently been adopted.^17,21−24^ At such temperatures, energy requirements and constraints on annealing media and furnace selection can be significantly reduced while still retaining fine-grained control over a relatively large range of diameters, lowering the barrier for entry for both similar research synthesis efforts and mass production. As reported herein, the unification of individual synthesis approaches combining synthetic control of the HSQ polymer cross-linking density within such lower temperature regimes brings with it the distinct advantages of both methodologies. This joint approach enables comparatively low-cost fabrication of highly crystalline Si NCs that display strong luminescence, remaining systematically tunable via variation in lower annealing temperatures across a broad range of NC diameters.

While bulk Si possesses an indirect bandgap resulting from the coupling between the conduction band minimum at momentum values corresponding to 0.8–0.85 of the X point and valence band maximum at Γ (often designated as Γ_25′_-Δ_1_),^16,25^ Si NCs of dimensions on the order of the Bohr radius are typically known to take advantage of the quasi-direct radiative HOMO–LUMO (highest/lowest occupied/unoccupied molecular orbital) transition resulting from significant quantum confinement.^14−17,26−28^ While confinement and its corresponding tunability effect on both emission and absorption have been the subject of rigorous investigation, very little has been suggested to reconcile the magnitude of variability (typically >∼1 eV) in reported absorption–emission energy difference, often labeled as apparent Stokes shift.^18,29,30^ Here, this often-overlooked behavior is examined by close scrutiny of the large dynamic range diffuse reflectance absorption (DRA) data afforded by such highly crystalline NCs in the context of the changing density of states (DOS) near the theoretical band edge and the associated links to quantum confinement. Commonly cited potential origins such as relative particle size polydispersity^30^ and the significant DOS decrease at photon energies near the emission energy^18,29^ are examined.

Although long-studied, precise establishment of the optically active transitions responsible for observed optical behaviors in confined Si has long been elusive, being still a subject of severe contention. Notwithstanding the potential pitfalls and inconsistencies often present in common analytical methods (such as Tauc, Cody, and regression analyses),^31,32^ additional difficulties can arise from variation in particle size,^14,30^*k*-vector blurring (and associated state mixing) with confinement,^14,33,34^ measurement-induced changes,^35^ and other nonidealities present in colloidal Si NC systems. Further inquest utilizing a confidence interval-based approach including multiple variations of Tauc analysis and other methods yields multiple distinct optical transitions with separation of those having direct, quasi-direct, and indirect natures. In every case examined herein, clear trends are apparent for the three distinct energy regimes. Observed behaviors in the low- (with close proximity to luminescence) and high-energy regions are attributed to confinement effects on both the indirect Γ_25′_-Δ_1_ and direct Γ_25′_–Γ_15_ transitions, respectively. In the midenergy area, two separate behaviors are evident: NCs with comparatively lower levels of confinement display correspondingly weaker absorption (identified here as Γ_25′_-L_1_), which are then overtaken by an increasingly more direct-like transition akin to those appearing from progressively higher proportions of Γ-X wave function mixing^33,34^ as confinement is increased. While the critical point for this changeover is fuzzy, previous works have postulated a range of ∼4–5 nm,^33,34^ consistent with that seen herein and near the oft-reported values for the Bohr radius of Si.^16^ Combined, these identifications allow for direct quantification of the relationships among the nature of transitions, DOS, and confinement within the Si NC material system.

## Experimental Methods

### Materials

Trichlorosilane (99%) was purchased from Sigma-Aldrich. 1-dodecene (96%) was purchased from Alfa Aesar. Hydrofluoric acid (48–51%, in water, HF) was purchased through Fisher or Acros. Common solvents such as methanol (99+%, MeOH), ethanol (anhydrous and 95%, EtOH), and toluene (99.5%) were ACS grade and purchased from Fisher or Acros. 1-dodecene was degassed using a freeze–pump–thaw technique performed three times before being stored under a N_2_ atmosphere. Methanol and ethanol were stored under molecular sieves and distilled prior to use. Toluene was dried with sodium and distilled prior to use.

### Synthesis of the HSQ Precursor

The HSQ polymer used in the synthesis of the Si NCs was produced based on a method previously reported in the literature.^17^ Under the N_2_ atmosphere, 80 mL of MeOH was added into a 250 mL flask, and a syringe was loaded with 4.5 mL HSiCl_3_ and capped in a sealed vial. The flask of MeOH was removed from the glovebox, placed in an ice water bath, connected to a Schlenk line, and continuously flushed with nitrogen. Under rapid stirring, the temperature of the system was cooled to 0–5 °C before the HSiCl_3_ was injected dropwise via syringe into the flask ensuring that the temperature never exceeded 10 °C. After the addition of HSiCl_3_, the solution was stirred for 5 min before 18 mL of deionized H_2_O was quickly injected, which rapidly increased the temperature to ∼30 °C. This solution was then stirred for 2 h, after which the newly formed gel was first rinsed five times with methanol using vacuum filtration and then stored under a vacuum overnight to remove any residual water and MeOH.

### Synthesis of Si NCs Embedded in a Silica Matrix

Si NCs embedded in a silica matrix were synthesized through thermal disproportionation. 0.4 g of the HSQ polymer was placed in a quartz boat, inserted into a tube furnace, and heated to 800–1100 °C, where it was held for 1 h with a ramp rate of 7 °C/min under a reducing gas mixture of Ar/H_2_ (95%/5%) having a flow rate of 20 mL/min. After 1 h of heating, the product was cooled at a rate of 2 °C/min, causing the white solid to change to a brown/black color, depending on the maximum annealing temperature.

### Liberation of Si NCs from the Silica Matrix

Etching was performed by using aqueous HF solutions to liberate phase pure Si NCs. *Caution: Solutions of HF are extremely hazardous and must be used in accordance with local regulations!* Neutralization of HF-containing waste or spills was performed with a solution of CaCl_2_. For this etching procedure, a 1:1:1 mL ratio of H_2_O:EtOH:HF was used for every 0.1 g of Si NCs in silica. First, ∼0.3 g of the Si NCs embedded in silica were ground into a powder using a mortar and pestle and added to a polypropylene centrifuge tube. Three milliliters of EtOH and H_2_O were added to the Si NCs in silica, and the tube was introduced into a N_2_ atmosphere where 3 mL of HF was added and stirred for 1 h. Directly after the addition of HF, the centrifuge tube was covered with aluminum foil to protect the etching solution from any ambient light. Almost immediately after the addition of HF, the suspension went from turbid brown/black to tan, indicating the liberation of the Si NCs from the silica matrix. To isolate the hydride-terminated NCs, 30 mL of toluene was added to the tube in 10 mL aliquots. The tube was vigorously shaken before the layers were allowed to separate, and the top organic layer was extracted into a clean centrifuge tube. If the etch was successful, the organic layer should appear as a turbid tan suspension, and the bottom, aqueous layer should be colorless and clear. The tube containing the organic layer (hydride-terminated Si NCs) was centrifuged at 7000 rpm for 5 min where the NCs formed a pellet at the bottom of the tube and the clear, colorless supernatant was removed.

### Surface Functionalization via Hydrosilylation

To further improve colloidal stability and protect the NC surface from oxidation,^14,20,35,36^ a thermal hydrosilylation was performed, replacing the hydride capping with a long-chain hydrocarbon. In an inert atmosphere, hydride-terminated Si NCs were dispersed in a three-neck flask containing 10 mL of 1-dodecene for every 0.3 g of annealed HSQ. The solution was attached to a Schlenk line where a N_2_ flow was introduced, and the temperature was increased to 190 °C. Within 5 min of heating, the turbid, tan suspension turned to an optically clear orange-red solution. This solution was heated overnight to allow for optimal surface passivation.

### Isolation and Purification

After the hydrosilylation, the NCs were isolated by adding 10 mL of toluene and then 30 mL of MeOH to the crude suspension followed by centrifugation at 6000 rpm for 30 min. The supernatant was removed, and 5 mL of toluene was added to the precipitate to redisperse the Si NCs. Then, 5 mL of MeOH was added to the Si NCs and centrifuged for 10 min to reprecipitate Si NCs. This procedure was repeated three times to purify the Si NCs.

### Characterization Procedures

Powder diffraction patterns were recorded using a PANalytical Powder X-ray diffractometer equipped with a Cu Kα (λ = 1.5418 Å) anode. The Scherrer formula was used to calculate the average crystallite size of the NCs.^37^ Raman spectra were recorded using a Thermo Scientific DXR Raman spectrophotometer with 532 nm laser excitation. Low-resolution TEM images were recorded using a Zeiss Model Libra 120 electron microscope operating at 120 kV. From these, average NC size and polydispersity (1σ) were obtained from a population of ∼200 particles per sample. High-resolution TEM (HRTEM) images and selected area electron diffraction (SAED) patterns were recorded by using a JEOL JEM-F200 cold FEG electron microscope operating at 200 kV. TEM grids were prepared by drop-casting a dilute solution of NCs in toluene onto a lacey-carbon-coated copper grid. EDX spectra were recorded using a Hitachi Model FE-SEM SU-70 scanning electron microscopy (SEM) operating at 15 keV with an *in situ* EDAX detector. Samples were prepared for SEM by transferring a small amount of NCs onto carbon tape and then sticking the carbon tape onto an aluminum holder. Solid-state diffuse reflectance spectra of the NCs were recorded using a Cary 6000i UV–vis-NIR spectrophotometer in double beam mode with an internal diffuse reflectance DRA 2500 attachment and BaSO_4_ background holder. The Kubelka–Munk remission function was used to convert measured reflectance to absorption data.^38−40^ Photoluminescence (PL) spectra of the drop-cast samples were measured by utilizing a Kimmon HeCd laser (325 nm/3.81 eV, ∼15 W/cm^2^) in conjunction with liquid nitrogen-cooled CCD and InGaAs detectors mounted onto a 30-cm focal length spectrograph. Emission spectra were corrected for the standard spectral response of the measurement system obtained via Quartz Tungsten Halogen (QTH) and Hg(Ar) calibration lamps. Samples were held at room temperature during the collection of all-optical data.

## Results and Discussion

### Physical Characterization

**Figure 1:**
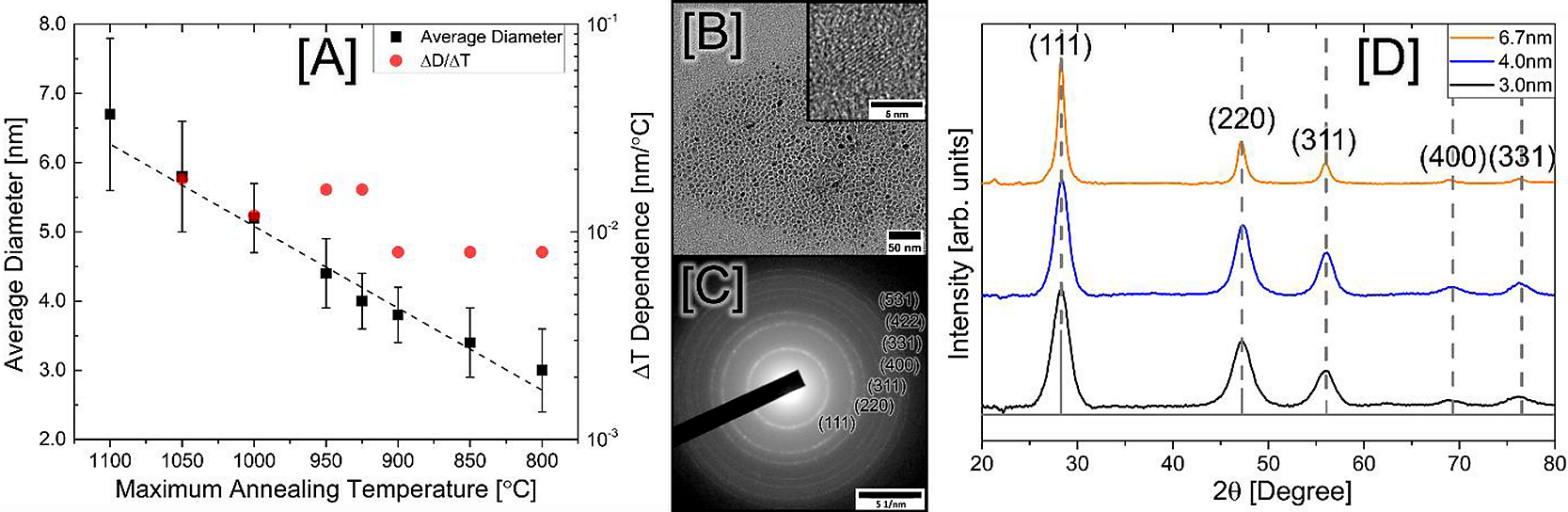
(A) Si NC diameters and polydispersity (obtained via TEM, black) plotted as a function of maximum annealing temperature. A dotted linear trendline is included to highlight the systematic reduction in NC size observed with corresponding temperature decrease. Change in diameter with temperature (Δ*D*/Δ*T*, red) is shown on the secondary axis. (B) Low-resolution bright-field TEM image of the largest Si NCs (6.7 nm, 1100 °C), with HRTEM highlighting the crystal lattice shown as an inset. (C) Representative SAED image of Si NCs exhibiting high crystallinity with lower indices marked. (D) Powder X-ray diffraction (PXRD) patterns of Si NCs were annealed at 1100 °C (orange), 925 °C (blue), and 800 °C (black). The ICCD-PDF overlay of diamond cubic Si (JCPS 00-001-0971) is also shown, with peak positions extended as vertical dashed lines.

As commercial HSQ has been reported to produce considerably smaller NCs even in higher temperature ranges (typically 1100–1400 °C),^15,17−20^ a maximum annealing temperature of 1100 °C was used for the synthesized HSQ-based Si NCs reported herein to serve as a baseline for comparison of physical, structural, and optical properties. To adjust the NC diameter, the temperature was reduced to 50 or 25 °C intervals. As displayed in Figures 1A, S1, and S2 (tabulated within Table S1 in the Supporting Information), resultant average diameters measured from TEM analysis were 6.7 ± 1.1 (1100 °C), 5.8 ± 0.8 (1050 °C), 5.2 ± 0.5 (1000 °C), 4.4 ± 0.5 (950 °C), 4.0 ± 0.4 (925 °C), 3.8 ± 0.4 (900 °C), 3.4 ± 0.5 (850 °C), and 3.0 ± 0.6 nm (800 °C), with an ostensibly linear trend of diameter reduction with decreasing maximum annealing temperature. This relatively consistent change in size corresponding to the systematic reduction in temperature (Δ*D*/Δ*T*) can be seen alongside the red points in Figure 1A. In some cases, as smaller diameters were reached, TEM imaging proved to be challenging due to the low contrast and size, with TEM images for all studied annealing temperatures displayed in Figure S1. For the largest set of NCs (6.7 nm, 1100 °C), low-resolution TEM (Figure 1B), HRTEM (Figure 1B inset), and SAED (Figure 1C) analyses display high degrees of crystallinity, which can be indexed to the diamond cubic crystalline structure of Si.

To assess any potential impact of annealing temperature on crystallinity, several selected samples were further analyzed using PXRD. As shown in Figure 1D, the Si NCs studied exhibited intense, broad Bragg reflections corresponding to nanocrystalline (111), (220), (311), (400), and (331) planes of diamond cubic silicon. No impurity peaks corresponding to SiO_2_ were observed, confirming the successful removal of the silica matrix and complete passivation of the NC surface with dodecane ligands. Additionally, the largest NCs were observed to exhibit the sharpest peaks. In contrast, the smaller NCs had intense, but wider peaks, indicating a smaller crystallite size while retaining high crystallinity. This suggests that lower annealing temperatures effectively decrease the NC size while retaining high crystallinity at all annealing temperatures used here, reinforced by the nonexistence of significant residual amorphous Si–Si bonding confirmed by supplemental Raman spectroscopy shown in Figure S3, further explored in the Supporting Information. Quantification of the observed peak broadening via the Scherrer equation can then be used to obtain a general estimate of NC crystallite size.^18,37^ Resulting analysis of the patterns presented in Figure 1D (recorded in Table S1) is in close agreement (within ±0.2 nm) with those obtained from the TEM analysis.

### Optical Properties

**Figure 2:**
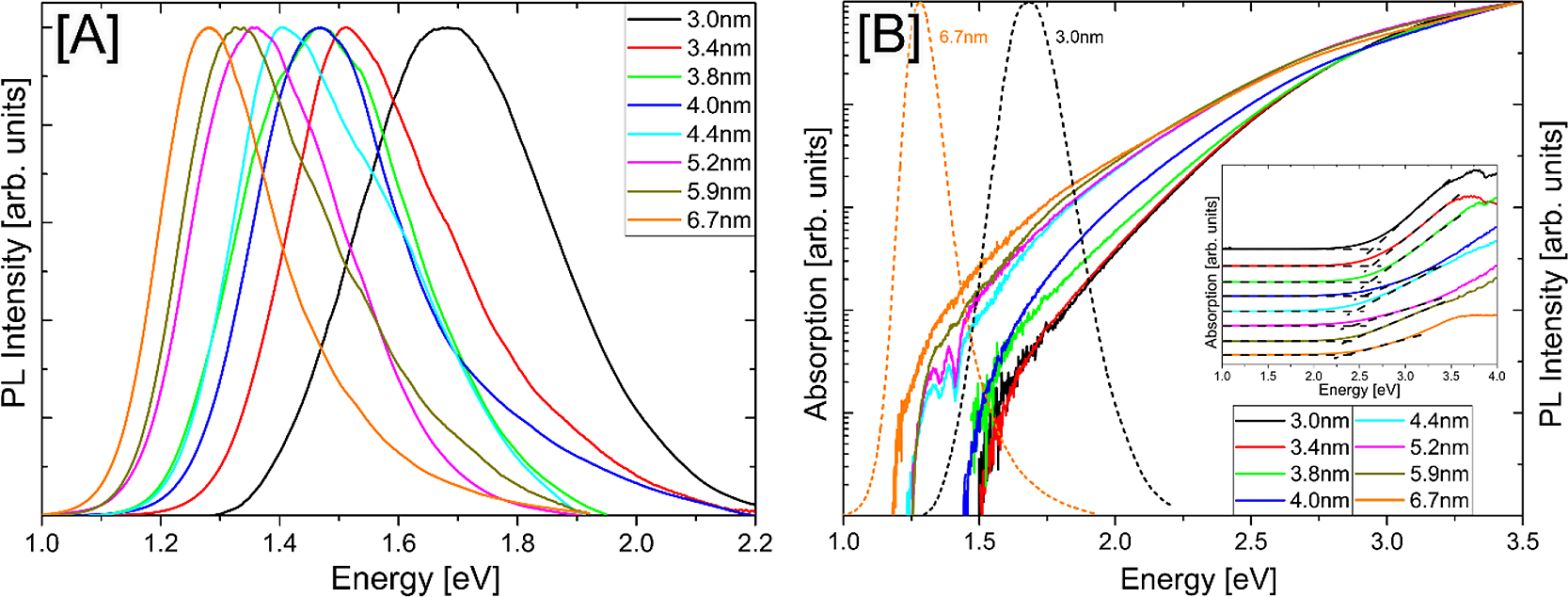
(A) Normalized room-temperature PL spectra of solid-state Si NC samples dispersed on Si. (B) Logarithmic-scale absorption spectra (normalized at 3.5 eV) from Kubelka–Munk processing of diffuse reflectance were measured from the same samples. PL spectra for the smallest (3.0 nm, 800 °C) and largest (6.7 nm, 1100 °C) are inlaid as black and orange dashed curves, respectively. Linear-scale absorption spectra (with arbitrary vertical offset) and representative linear regression lines are shown in the inset.

The ability of low-temperature annealing of HSQ polymers to provide highly crystalline Si NCs with a wide range of obtainable diameters positions them as ideal candidates for systematic probing of band structure and the resultant optical properties. Steady-state PL spectra obtained from NC samples of various sizes are shown in Figure 2A. Spanning from the visible into the NIR with peak energies between 1.29 and 1.68 eV, these emission spectra also correlate well with increasing NC diameter and the associated variation in maximum annealing temperature across the 6.7–3.0 nm range, providing additional strong evidence of being quantum confinement-related. Comparison of peak emission energies at each relative size obtained from the dodecane-capped Si NCs synthesized at low temperatures in this study to analogous structures reported elsewhere being created using more traditional higher temperature methods finds commensurate trends,^14,15,20^ indicating that the proposed low-temperature technique may serve as an adequate alternative. It is important to note that minor atmospheric exposure during measurements can potentially lead to photo-oxidation^35,41^ (especially given the inherently large surface-to-volume ratio), and the observed peak energies might be argued to show some similarity to those attributed to oxygen-related surface defect states.^28,35^ However, the relative stability of alkyl-capping to oxidation and reported comparisons with NCs having various passivation methodologies (including 1-dodecene, as used here) strongly suggests that the observed red-to-NIR emission is related to the quantum-confined core.^14,42^ Gaussian fits to the displayed peaks provide relatively wide fwhm values averaging ∼310 meV, a property likely stemming from the aforementioned particle size polydispersity.^20,30,43^ These values are compared directly in Figure S4A in the Supporting Information. Notably, in the cases of the largest NC emitters (at 5.9–6.7 nm diameters), the respective emission spectra were observed to become decidedly non-Gaussian on the low-energy side. Similar behavior as emission energies approach the bulk Si bandgap has been previously reported for comparable alkyl-passivated Si NC systems (albeit directly attributed to diameter as opposed to emission energy); potentially an indicator of reabsorption or a “fundamental” low-energy limit of bulk Si for this class of NCs, as diameters trend away from more confined values.^18,20^

Parallel trends to the emission discussed above are seen in the experimental absorption inset displayed in Figure 2B. Absorption spectra were obtained from diffuse reflectance measurements by using the Kubelka–Munk remission function:
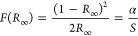
1

containing the reflectance parameter  and scattering coefficient *S* (here assumed to be wavelength independent, thus *F*(*R*_∞_) ∝ α).^38−40^ In the linear scale, the pronounced absorption onsets were found by least-squares linear regression analysis^39,44^ across the full measured range to be within 2.36–2.66 eV, correspondingly increasing with decreasing Si NC diameter from 6.7 to 3.0 nm. While NCs on the larger end of this size range are generally considered bulk-like (indirect gap),^29,45^ this systematic blueshift with shrinking size indicates some dependence on increasing levels of quantum confinement.

### Apparent Energy Shifts

Direct comparison of experimental PL peaks (Figure 2A) and linear regression-obtained absorption onsets (Figure 2B inset, recorded in Table S2), however, reveals inconsistent large differences in energy shift (Δ*E*) averaging 1.07 eV, with similarly large Δ*E* values being reported across the literature.^18,30,43^ This echoes the infamous difficulty of measuring meaningful absorption onsets in indirect nanocrystalline systems such as Si, where no sharp excitonic peaks or defined critical points appear in comparison to those seen in commonly studied direct bandgap NCs such as CdSe^46^ or metal halide perovskites.^47^ Notably, such featureless absorption spectra have been reported even in quasi-direct alkyl-terminated Si QDs (on the order of ∼2.0 nm) purportedly exhibiting phonon-less recombination dynamics,^35^ further muddying the waters. Numerous attempts have been made to explain this intriguing behavior, one more widely accepted theory of which was proposed by Kovalev et al. to be an apparent Stokes shift; effectively an artifact produced by the behaviorally significant decrease in DOS as photon energies approach the band edge.^18,29^

This reliance on DOS (as opposed to typical absorption edge and Stokes shift quantification^26,29^) can be further explored in the low-absorption domain by visualizing the normalized absorption spectra on a logarithmic scale, as illustrated in Figure 2B. The anomalous valleys located at 1.36 and 1.41 eV visible in some spectra (shown in Figure S4B in the Supporting Information) are byproducts of background correction using the toluene solvent carrier reference,^48^ and result in negligible average energy shifts of <0.17% when removed via locally weighted linear regression smoothing. In this regime, changes in the slope and relative absorption separation are clearly revealed, particularly at the comparatively low energy sides of the spectra (<∼2 eV) approaching the PL emission edges. For example, relative absorption slopes within the vicinity of each respective onset average are almost 3× lower for the smaller subset of Si NCs (≤4.0 nm), compared to those obtained from NCs with diameters ≥4.4 nm nearing the more bulk-like size regime. Qualitatively, this decrease can be described as a “softer”, more gradual onset as quantum confinement is increased. This reduction has sometimes been attributed singularly to broadening resulting from the NC size distribution, given a constant degree of polydispersity with respect to each of the compared NC diameters.^30,43^ While this is acknowledged as a probable contributor, the consistency of this trend even alongside significant variance in polydispersity (±0.4–1.1 nm) across the range of synthesized NC sizes suggests additional factors may be involved. One intuitive behavior appearing consistent with the observed trends is the spreading of discrete energy levels within the progressively more confined NCs as the diameter is reduced. The consequent expansion between adjacent energy levels results in the further lengthening of the DOS relative to confinement, realized as an increasingly stretched extension of the apparent absorption onset.^26^ This is further supported by prominent changes in Raman line shape (shown in Figure S3 in the Supporting Information) in the vicinity of the aforementioned ∼4.0/4.4 nm crossover point; potentially an additional indicator of the systematic increases in intraband quantum level spacing predicted here and elsewhere.^49,50^

### Band Structure and Transitions

**Figure 3:**
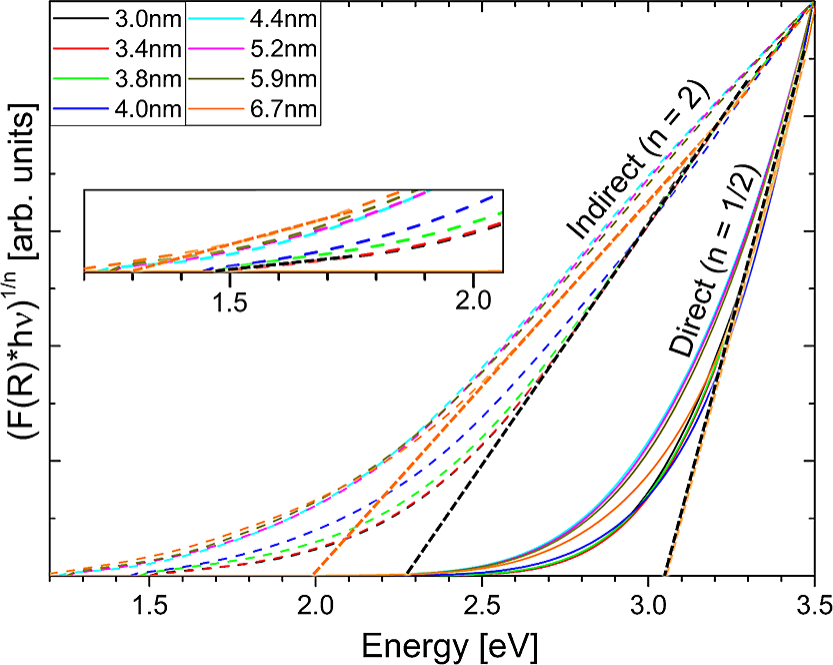
Normalized Tauc curves for Si NC samples of all of the synthesized diameters. Solid and dashed curves represent allowed direct (*n* = 1/2) and allowed indirect (*n* = 2) transitions,^51^ respectively. Demonstrative extrapolations relating to each transition of interest are shown as black and orange dotted lines, corresponding to the smallest (3.0 nm, 800 °C) and largest (6.7 nm, 1100 °C) Si NCs. The low-energy portion of the plot is magnified in the inset.

Further numerical analysis of derived absorption data (recorded in Table S3) provides significantly more insight into the bandlike structures present in the synthesized NCs. Examination of absorption through Tauc analysis is shown in Figure 3. Processing via the Tauc equation
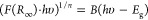
2where *F*(*R*_∞_) ∝ α as discussed above, photon energy is given as *h*υ, *n* represents a variable transition factor (*n* = 1/2 for allowed direct and *n* = 2 for allowed indirect transitions), and *E*_g_ is the extracted Tauc energy gap (with *B* as a multiplicative prefactor representing “relative strength” of the transition corresponding to factors such as oscillator strength and DOS),^27,40,51^ can often be helpful in separating and quantifying the direct-like and indirect transitions contributing to absorption. In this vein, it is immediately clear from Figure 3 that extrapolation to obtain single overall energy gap descriptions will not be sufficient to fully describe the observed trends. Similar drawbacks have been noted in previously published reports suggesting the presence of multiple evident transitions within confined Si nanostructures, for which discrete Tauc analysis of processed absorption data can be performed within the respective energy ranges corresponding to the approximate linear regions of each involved transition.^27,52,53^ Identification of these linear regions is not always straightforward, especially in cases with significant variance in DOS/strengths of transitions-of-interest, wide onsets caused by line width broadening, or broken symmetry allowed interband and critical point transitions resulting from band intermixing. By the confidence interval-based approach utilized here, however, extrapolation via calculated Tauc curves from three carefully selected wide linear regions in addition to all-encompassing multiline piecewise fits preserves both sensitivity to weakly expressed absorption nearby to the PL energy and the impartiality required to provide significant reductions in processing variability. Application of alternative methodologies such as arbitrarily defined 50% absorption onsets and the recently described Boltzmann sigmoidal function fitting process,^32^ although providing additional support to those energies found from Tauc analyses, by themselves do not completely alleviate these concerns when applied to the α(*E*) measured here; the former again recording a single onset with large Δ*E* values and the latter, while capable of reliably identifying separate transitions in the mid- and high-energy regions, lacks notable features in the proximity of the observed radiative transition. Methodological comparisons with all derived energy gaps can be seen in Figure S5 and are tabulated in Tables S3 and S4 in the Supporting Information.

**Figure 4:**
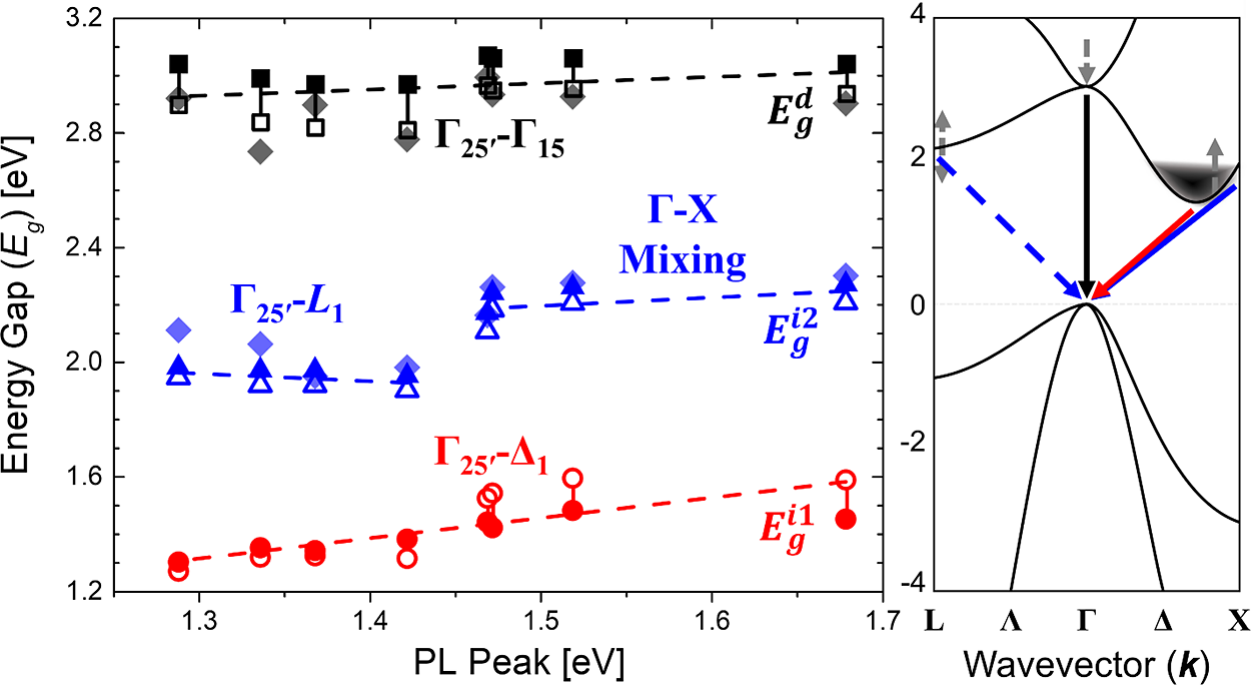
Dependence of direct (*E*_g_^d^, black) and indirect (*E*_g_^i1^ and *E*_g_^i2^, red and blue) Tauc gaps on PL peak energy as measured from Si NCs across the full range of synthesized diameters. Manual (filled symbols) and multiline piecewise (open symbols) fitted Tauc extrapolated gaps are expressed as connected confidence intervals with corresponding Boltzmann function fit energies demarcated by filled diamond symbols. Dashed linear trendlines are provided as a guide for each respective data set. An illustrative Si NC band diagram displaying the effects of increasing confinement (gray arrows) and transition attributions (colored arrows) is included alongside (patterned after refs (14 and 25)).

As discussed above, the indirect (*n* = 2) Tauc curves obtained from the variously sized Si NCs (seen as dotted curves in Figure 3) can then be further split according to the multiple linear regions present, allowing supplementary fine-grained analysis of the respective recombination mechanisms. Extrapolated energy gaps within the low-energy region of 1.50–1.75 eV are shown in Figure 4, spanning *E*_g_^i1^ = 1.30 (1.27)–1.48 (1.59) eV (multiline piecewise fits in parentheses) and increasing linearly with decreasing NC diameter. The consistently close proximity (averaging <60 meV) and distinct trend matching of *E*_g_^i1^ to respective PL peaks highly suggest the correlation of this transition to the observed tunable red emission. A mixture of size polydispersity, low DOS near band edge, and an increase in direct-like nature of relatively smaller particles (<∼4.0 nm) may be considered likely culprits for the slight discrepancies between PL and absorption. This is further strengthened when relative absorption strength is taken into account, with prefactor *B*^i1^ roughly halving for NCs smaller than this threshold. Although the exact identification of this core-related transition is debated as discussed above, it is commonly ascribed to the indirect Γ_25′_-Δ_1_ gap.^14,27,42^ Despite the continuing attributional disagreements as to the origins of these optical transitions, the lack of defined Stokes shift between the radiative and absorbing states is consistent with Kovalev et al.’s interpretation.^29^

Extrapolated indirect gaps inside of the midenergy region (2.50–3.25 eV) are analogously characterized by the relatively consistent linear trend shown in Figure 4, tracking *E*_g_^i2^ = 1.95 (1.90)–2.27 (2.21) eV following increasing confinement within the Si NCs and their associated PL blueshift. Energy values derived from Boltzmann function fitting also indicate analogous indirect gaps from 1.95 to 2.30 eV. However, in this intermediate regime, differences again appear depending on the NC size range being examined. For NCs with diameters >4.0 nm, indirect energy gaps and prefactor ratios *B*^i2^/*B*^i1^ remain similar, averaging *E*_g_^i2^ ≈ 1.97 (1.92) ± 0.02 eV and *B*^i2^/*B*^i1^ ≈ 4.2 ± 0.3, respectively. While these values are comparable to those reported for Γ_25′_-L_1_ transitions,^27,54^ it is apparent that NCs at and above this size range lack significant quantum confinement. Within this context, the behavior of NCs having diameters ≤4.0 nm becomes more complex, particularly when the oft-reported reciprocal space “fuzziness” of confined Si is considered.^14^ For these, progressive increments of both *E*_g_^i2^ = 2.17 (2.11)–2.27 (2.21) eV and *B*^i2^/*B*^i1^ = 6.9–13.6 are potentially a consequence of increasing confinement, bringing this transition nearer to the quasi-direct regime. A theory further supported by both the sudden onsets seen from the direct Tauc curves in Figure 3 and recent observations of strong absorption enhancement near ∼2.3 eV in related systems being identified as mixing of the direct Γ wave function into indirect X core states.^14,33−35^ Though this nominally forbidden mixing may be faintly present in weakly confined NCs, further reduction in diameter and the confinement-induced relaxation of momentum conservation rules begets coupling of increasingly larger proportions of Γ character into X states,^33,34^ dramatically growing absorption correspondingly until all other potential contributions within this regime are overtaken.

For the strong but comparatively featureless direct (*n* = 1/2) Tauc curves (Figure 3, solid curves), extrapolation of the linear regions between 3.25 and 3.45 eV gives a consistent average of *E*_g_^d^ ≈ 3.02 (2.90) ± 0.05 (0.09) eV, having little variance across the NC size range (as illustrated in Figure 4) and corresponding closely to both the onset values at 50% of maximum absorption and those obtained from Boltzmann fitting (averaging 3.01 ± 0.08 and 2.88 ± 0.15 eV, respectively) of the absorption spectra in Figure 2B. This direct-like transition resides in the nearby range of both the bulk Si direct gap (3.4 eV) and the often-reported fast blue PL, pointing toward its likely association with core-related direct Γ_25′_-Γ_15_ transitions.^14,27,33,54^ While it should be additionally noted that the measured Tauc onsets for comparatively smaller NCs (≤4.0 nm) appear “sharper” and more direct-like with respect to those above this critical diameter, distinct classification of this quasi-direct crossover point is similarly highly debated and is liable to be dependent upon numerous additional factors (including particle diameter, surface passivation, and crystalline quality).^14,15,30,33,43,49^

## Conclusions

Using a comparatively low-temperature modification of the traditional high-temperature HSQ disproportionation method, dodecane-capped colloidal Si NCs were synthesized within locally produced HSQ polymer matrices having resultant diameters tunable between 3.0 and 6.7 nm by variation of maximum annealing temperature between 800 and 1100 °C, respectively. By a combination of structural characterization techniques including TEM, SAED, and PXRD, these NCs were found to be highly crystalline and exhibit relatively narrow-size polydispersity; comparable to NCs fabricated via conventional high-temperature methods.

Strong PL emission was observed for all samples, with peaks ranging from 1.29 to 1.68 eV with correspondingly decreasing NC diameter, consistent with both the size tunability expected as a result of increasing quantum confinement and values previously reported in the literature for similar systems.^14,15,18,20^ Comparison with absorption onsets obtained from traditional linear regression of the converted absorption (DRA) spectra, which additionally followed the expected blueshift trend with increasing confinement, suggest large apparent Stokes shifts on the order of ∼1.0 eV. Further evaluation of the NC absorption spectra, both qualitatively and in discrete energy spans via indirect/direct Tauc analysis, concluded that this apparent Δ*E* is likely an artifact of the vanishingly low DOS inherent in significantly confined indirect systems at energies approaching the emitting states.^29^ Various methods of quantification for the several discrete linear regions present within the Tauc curves revealed three distinct transitions, separately designated as diameter-tunable indirect Γ_25′_-Δ_1_ in the close vicinity of PL (∼1.30–1.48 eV), indirect Γ_25′_-L_1_ (NC diameters ≥∼4.4 nm) being overtaken (for NC diameters ≤∼4.0 nm) by confinement-dependent quasi-direct mixing of Γ-X wave functions in the midenergy regime (∼1.95–2.27 eV), and relatively diameter-independent direct Γ_25′_–Γ_15_ at higher energy values approaching ∼3 eV. Together, these indicate that the Si NCs synthesized via this low-cost and low-temperature HSQ polymer methodology possess high crystalline quality, with analysis of their prominent absorption and emission components permitting the identification of numerous evident transition- and confinement-related tuning parameters, uncovering additional still-unknowns in this long-studied material system and pointing toward its vast potential within future optoelectronic, *in*/*ex vivo*, and photovoltaic applications.

## Supporting Information Available

TEM images and size histograms of all Si NC samples, measured Raman spectra with brief discussion, plots of fwhm and polydispersity, supplemental plots of absorption and fit methodology, and tabulated data of observed physical and optical properties (PDF)
