# Supplementary material for: Size-Tunable Band Structure and Optical Properties of Colloidal Silicon Nanocrystals Synthesized via Thermal Disproportionation of Hydrogen Silsesquioxane Polymers
Source: J Phys Chem C Nanomater Interfaces. 2024 Jun 17;128(25):10483–91. doi: 10.1021/acs.jpcc.4c01462 (PMC11215768; doi:10.1021/acs.jpcc.4c01462)
Supplement: Supplementary file 1 — jp4c01462_si_001.pdf [file jp4c01462_si_001.pdf]

Size-Tunable Band Structure and Optical Properties of Colloidal Silicon Nanocrystals Synthesized via Thermal Disproportionation of Hydrogen Silsesquioxane Polymer

David S. Pate,[1] Griffin C. Spence,[2] Lisa S. Graves,[2] Indika U. Arachchige,[2] and Ümit Özgür[1]*

[1]Department of Electrical and Computer Engineering, Virginia Commonwealth University, Richmond, Virginia 23284-9052, USA

[2]Department of Chemistry, Virginia Commonwealth University, Richmond, Virginia 23284-9059, USA

*Corresponding Author: uozgur@vcu.edu

Supporting Information

PHYSICAL CHARACTERIZATION.

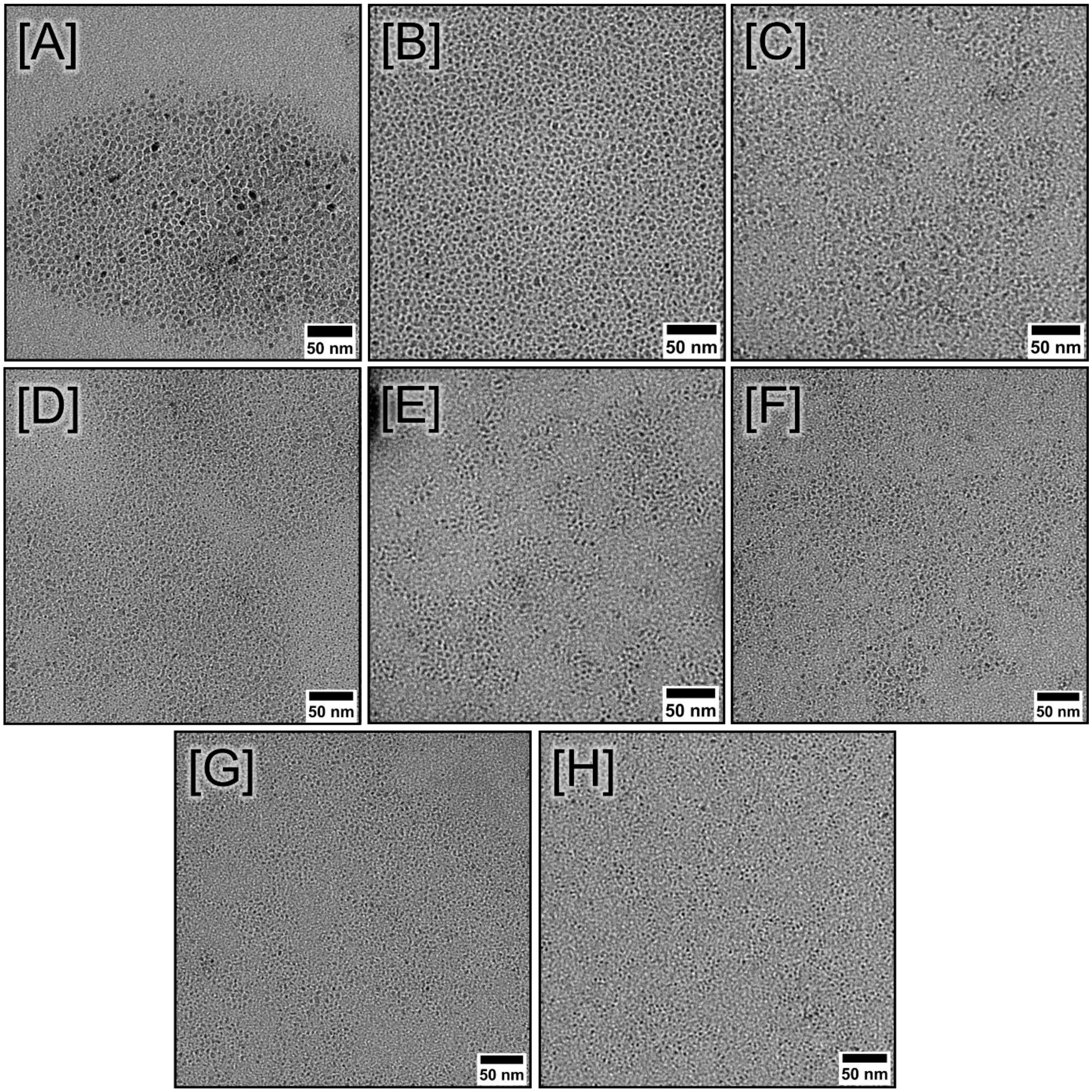

Figure S1. Low-resolution brightfield TEM images of Si NCs annealed at **(A)** 1100 °C (also seen in Figure 1A), **(B)** 1050 °C, **(C)** 1000 °C, **(D)** 950 °C, **(E)** 925 °C, **(F)** 900 °C, **(G)** 850 °C, and **(H)** 800 °C.

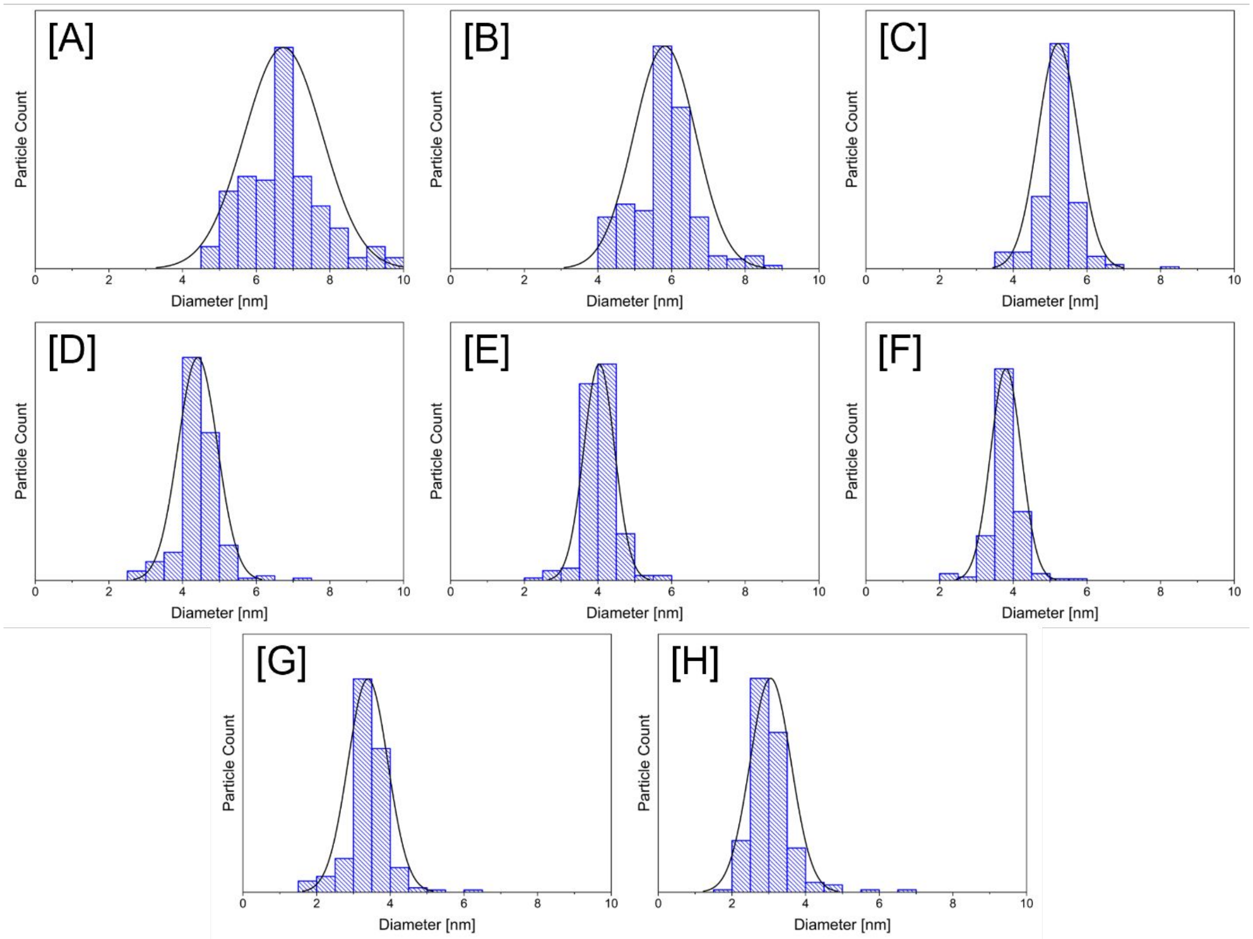

Figure S2. Size histograms of Si NCs produced at **(A)** 1100 °C, **(B)** 1050 °C, **(C)** 1000 °C, **(D)** 950 °C, **(E)** 925 °C, **(F)** 900 °C, **(G)** 850 °C, and **(H)** 800 °C. Average particle diameters and size dispersity values (1σ) were calculated from TEM images of ~200 particles per sample.

Table S1. Experimental and physical characteristics of synthesized Si NCs.

Sample	Annealing Temperature [°C]	Crystallite Size (XRD) [nm]	Diameter (TEM) [nm]	Polydispersity (TEM) [nm]
**A**	1100	6.5	6.7	1.1
**B**	1050		5.9	0.8
**C**	1000		5.2	0.5
**D**	950		4.4	0.5
**E**	925	3.9	4.0	0.4
**F**	900		3.8	0.4
**G**	850		3.4	0.5
**H**	800	2.9	3.0	0.6

RAMAN SPECTROSCOPY.

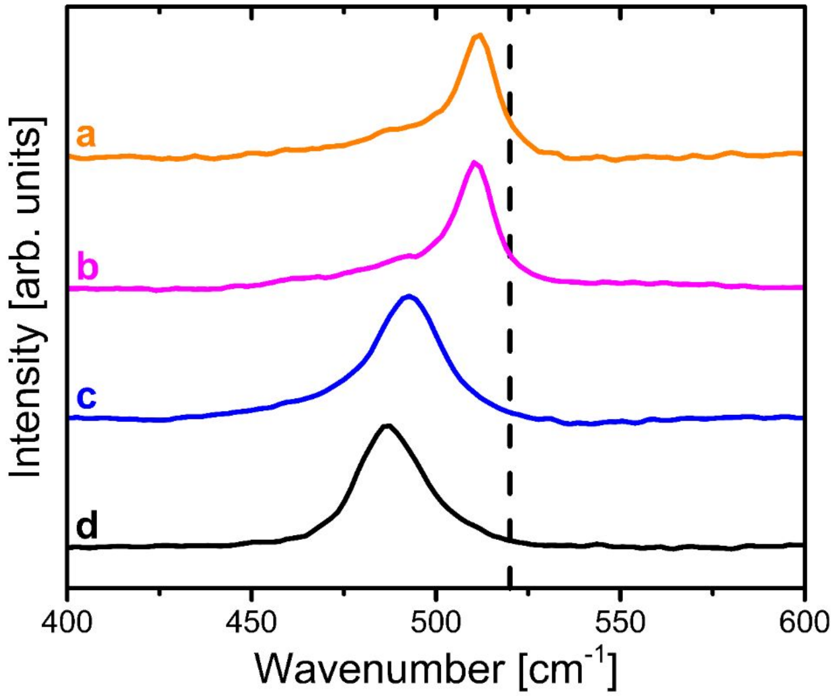

Figure S3. Raman spectra of Si NCs with experimental diameters **(a)** 6.7 nm (1100 °C), **(b)** 5.2 nm (1000 °C), **(c)** 4.0 nm (925 °C), and **(d)** 3.0 nm (800 °C). The bulk silicon Si-Si phonon mode (520 cm^{-1}) is shown as a dashed vertical line for reference.

Raman spectroscopy was additionally employed, both to further explore the high degree of crystallinity and investigate phonon modes of the local bonding environment present across a representative selection of Si NC diameters (shown in Figure S3). Compared to the peak at 520 cm^{-1} corresponding to the crystalline Si-Si bonds in bulk (dashed line in Figure S3),[1,2] increasing redshift and broadening of the Si-Si peak is observed with decreasing crystallite size. The Si-Si peak shifted consistently from 511.4 cm^{-1} for the largest NCs to 486.6 cm^{-1} for the smallest NCs. The main contributing factor to peak position shift and tailing effect is likely phonon-confinement in nanocrystalline silicon,[3,4,5] due to an increased ratio of undercoordinated Si atoms towards the

surface of the nanocrystal. As nanocrystal size decreases, the surface energy will inherently increase due to the amount of undercoordinated atoms, leading to the apparent tailing and broadening effects. Such progressive asymmetry can also be examined in the context of confined electron-phonon interactions, where varying lineshapes are observed in relation to NC size.[6] The crossover, where intraband quantum level spacing reaches the LO phonon energy (~65 meV) and the electron-LO phonon interaction is enhanced, is reported to occur for NC diameters < ~5 nm,[7] consistent with the data in Figure S2 for the smaller 3.0 and 4.0 nm NCs. It should be noted that these NCs did not exhibit any broad amorphous Si-Si band, which has been reported at 480 – 475 cm^{-1},[1,2] particularly in smaller (≤ ~4 nm) Si NCs produced with HSQ and is attributed to increased amorphized Si-Si bonds as annealing temperature decreases.[4,8] This suggests that the synthesis reported here largely avoids amorphization even at annealing temperatures as low as 800 °C.

OPTICAL CHARACTERIZATION.

Quantum yield (QY) estimates utilizing comparison of photoluminescence intensities obtained from the smallest 3.0 nm NCs and a Rhodamine 6G standard indicate a tentative QY in the range of ~0.15 – 0.30. However, it should be noted that the absolute value of QY will not impact identification of the optical transitions investigated here.

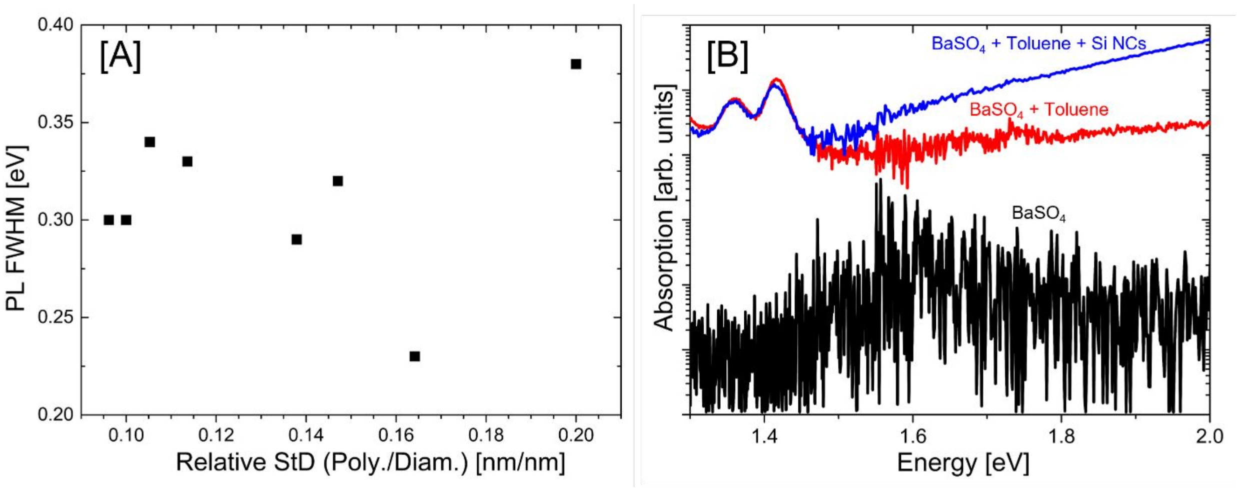

Figure S4. [A] Plot of PL FWHM compared to relative standard deviation (polydispersity divided by average diameter) of corresponding Si NCs. **[B]** Comparative plot of measured absorption with $BaSO_4$ substrate only (black), $BaSO_4$ with toluene (red), and Si NCs dispersed in toluene and drop-cast on $BaSO_4$ (blue).

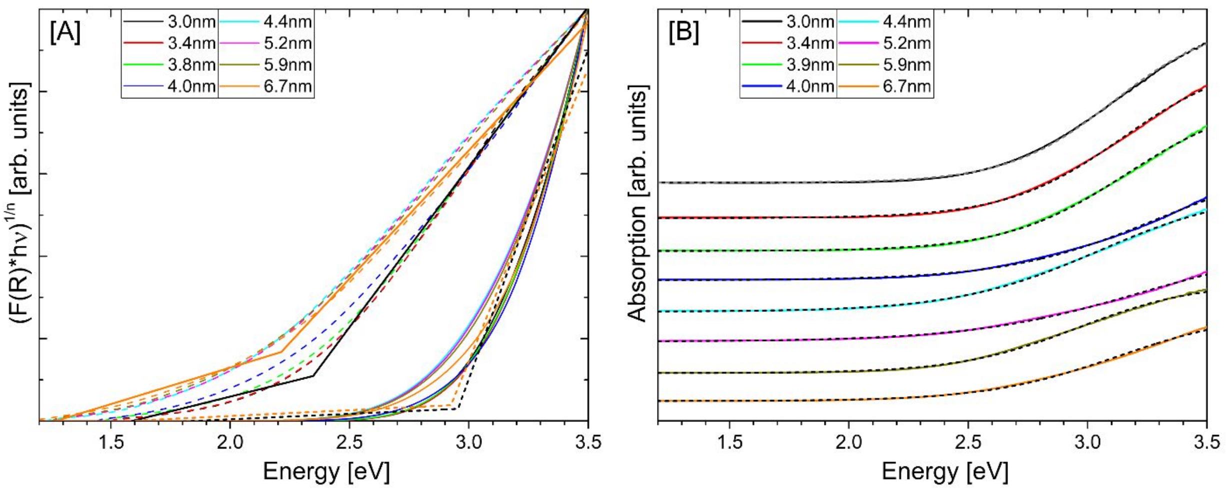

Figure S5. Illustrative fitting results for **[A]** multiline piecewise fits to indirect/direct (dotted/solid respectively) normalized Tauc curves shown for the smallest (3.0 nm, 800 °C, black) and largest (6.7 nm, 1100 °C, orange) Si NCs in each case, and **[B]** Boltzmann sigmoidal fits (dotted curves) overlaid onto all Si NC absorption spectra (solid lines, normalized and vertically offset for clarity). Boltzmann factors of $n_I^{Boltz} = 3.6$ and $n_D^{Boltz} = 0.9$ were utilized to determine corresponding indirect and direct energy gaps.[9]

Table S2. Direct comparison of various optical parameters measured from synthesized Si NCs.

Sample	PL Peak [eV]	PL FWHM [eV]	Absorption Onset (DRA) [eV]	ΔE [eV]
**A**	1.29	0.23	2.36	1.07
**B**	1.34	0.29	2.39	1.05
**C**	1.37	0.30	2.42	1.05
**D**	1.42	0.33	2.44	1.02
**E**	1.47	0.30	2.56	1.09
**F**	1.47	0.34	2.63	1.16
**G**	1.52	0.32	2.64	1.12
**H**	1.68	0.38	2.66	0.98

Table S3. Comparison of direct and indirect energy gaps (given in eV) extracted from Si NC DRA spectra and Tauc curves via multiple processing methodologies.

	50% Abs.	Boltzmann			Manual Extraction				Multiline Piecewise		
**Sam.**	E_g^d	E_g^d	E_g^{i2}		E_g^d	E_g^{i2}	E_g^{i1}		E_g^d	E_g^{i2}	E_g^{i1}
**A**	3.01	2.92	2.11		3.04	1.98	1.30		2.90	1.95	1.27
**B**	2.96	2.73	2.06		2.99	1.97	1.35		2.84	1.92	1.32
**C**	2.94	2.90	1.95		2.97	1.97	1.34		2.82	1.92	1.32
**D**	2.93	2.78	1.98		2.97	1.95	1.38		2.81	1.90	1.31
**E**	3.07	2.99	2.16		3.07	2.17	1.44		2.97	2.11	1.52
**F**	3.06	2.93	2.26		3.06	2.24	1.42		2.95	2.18	1.54
**G**	3.06	2.92	2.28		3.06	2.26	1.48		2.95	2.21	1.59
**H**	3.05	2.90	2.30		3.04	2.27	1.45		2.94	2.21	1.59

Table S4. Comparison of calculated energy gap fitting error (given in meV) and associated R^2 *goodness of fit* values for each applicable analysis approach.

	**Boltzmann**			**Manual Extraction**				**Multiline Piecewise**		
**Sam.**	E_g^d	E_g^{i2}		E_g^d	E_g^{i2}	E_g^{i1}		E_g^d	E_g^{i2}	E_g^{i1}
**A**	4 (0.999)	4 (0.999)		4 (0.997)	2 (0.999)	2 (0.995)		4 (0.979)	3 (0.997)	4 (0.997)
**B**	2 (0.999)	2 (0.999)		4 (0.998)	< 1 (0.999)	2 (0.991)		4 (0.985)	3 (0.998)	5 (0.998)
**C**	6 (0.998)	6 (0.998)		4 (0.998)	1 (0.999)	1 (0.996)		3 (0.985)	2 (0.998)	4 (0.998)
**D**	3 (0.999)	3 (0.999)		4 (0.998)	1 (0.999)	2 (0.995)		3 (0.985)	2 (0.998)	5 (0.998)
**E**	6 (0.998)	6 (0.998)		4 (0.997)	3 (0.998)	1 (0.994)		4 (0.982)	3 (0.997)	6 (0.997)
**F**	3 (0.999)	3 (0.999)		4 (0.997)	< 1 (0.999)	< 1 (0.999)		3 (0.985)	3 (0.998)	7 (0.998)
**G**	2 (0.999)	2 (0.999)		4 (0.997)	< 1 (0.999)	< 1 (0.996)		3 (0.986)	3 (0.998)	7 (0.998)
**H**	2 (0.999)	2 (0.999)		4 (0.997)	1 (0.999)	1 (0.995)		3 (0.987)	3 (0.997)	8 (0.997)

REFERENCES.

(1) Yogi, P.; Tanwar, M.; Saxena, S. K.; Mishra, S.; Pathak, D. K.; Chaudhary, A.; Sagdeo, P. R.; Kumar, R. Quantifying the Short-Range Order in Amorphous Silicon by Raman Scattering. *Anal. Chem.* **2018**, *90* (13), 8123–8129. DOI:10.1021/acs.analchem.8b01352.

(2) Parker, J. H.; Feldman, D. W.; Ashkin, M. Raman Scattering by Silicon and Germanium. *Phys. Rev.* **1967**, *155* (3), 712–714. DOI:10.1103/PhysRev.155.712.

(3) Campbell, I. H.; Fauchet, P. M. The Effects of Microcrystal Size and Shape on the One Phonon Raman Spectra of Crystalline Semiconductors. *Solid State Commun.* **1986**, *58* (10), 739–741. DOI:10.1016/0038-1098(86)90513-2.

(4) Hessel, C. M.; Wei, J.; Reid, D.; Fujii, H.; Downer, M. C.; Korgel, B. A. Raman Spectroscopy of Oxide-Embedded and Ligand-Stabilized Silicon Nanocrystals. *J. Phys. Chem. Lett.* **2012**, *3* (9), 1089–1093. DOI:10.1021/jz300309n.

(5) Sautter, K. E.; Schuck, C. F.; Garrett, T. A.; Weltner, A. E.; Vallejo, K. D.; Ren, D.; Liang, B.; Grossklaus, K. A.; Vandervelde, T. E.; Simmonds, P. J. Self-Assembly of Tensile-Strained Ge Quantum Dots on InAlAs(111)A. *J. Cryst. Growth* **2020**, *533* (January), 125468. DOI:10.1016/j.jcrysgro.2019.125468.

(6) Sagar, D. M.; Atkin, J. M.; Palomaki, P. K. B.; Neale, N. R.; Blackburn, J. L.; Johnson, J. C.; Nozik, A. J.; Raschke, M. B.; Beard, M. C. Quantum Confined Electron–Phonon Interaction in Silicon Nanocrystals. *Nano Lett.* **2015**, *15* (3), 1511–1516. DOI:10.1021/nl503671n.

(7) Lee, S.; Lee, Y.; Song, E. B.; Hiramoto, T. Observation of Single Electron Transport via Multiple Quantum States of a Silicon Quantum Dot at Room Temperature. *Nano Lett.* **2014**, *14* (1), 71–77. DOI:10.1021/nl403204k.

(8) Terada, S.; Xin, Y.; Saitow, K. Cost-Effective Synthesis of Silicon Quantum Dots. *Chem. Mater.* **2020**, *32* (19), 8382–8392. DOI:10.1021/acs.chemmater.0c02320.

(9) Zanatta, A. R. Revisiting the Optical Bandgap of Semiconductors and the Proposal of a Unified Methodology to Its Determination. *Sci. Rep.* **2019**, *9* (1), 11225. DOI:10.1038/s41598-019-47670-y.
